# Next-Generation Sequencing in Post-mortem Genetic Testing of Young Sudden Cardiac Death Cases

**DOI:** 10.3389/fcvm.2016.00013

**Published:** 2016-05-30

**Authors:** Najim Lahrouchi, Elijah R. Behr, Connie R. Bezzina

**Affiliations:** ^1^Department of Clinical and Experimental Cardiology, Heart Center, AMC, Amsterdam, Netherlands; ^2^Cardiology Clinical Academic Group, St George’s University of London, London, UK

**Keywords:** sudden cardiac death, post-mortem genetic testing, molecular autopsy, next-generation sequencing, channelopathy, cardiomyopathy

## Abstract

Sudden cardiac death (SCD) in the young (<40 years) occurs in the setting of a variety of rare inherited cardiac disorders and is a disastrous event for family members. Establishing the cause of SCD is important as it permits the pre-symptomatic identification of relatives at risk of SCD. Sudden arrhythmic death syndrome (SADS) is defined as SCD in the setting of negative autopsy findings and toxicological analysis. In such cases, reaching a diagnosis is even more challenging and post-mortem genetic testing can crucially contribute to the identification of the underlying cause of death. In this review, we will discuss the current achievements of “the molecular autopsy” in young SADS cases and provide an overview of key challenges in assessing pathogenicity (i.e., causality) of genetic variants identified through next-generation sequencing.

## Introduction

**Figure 1:**
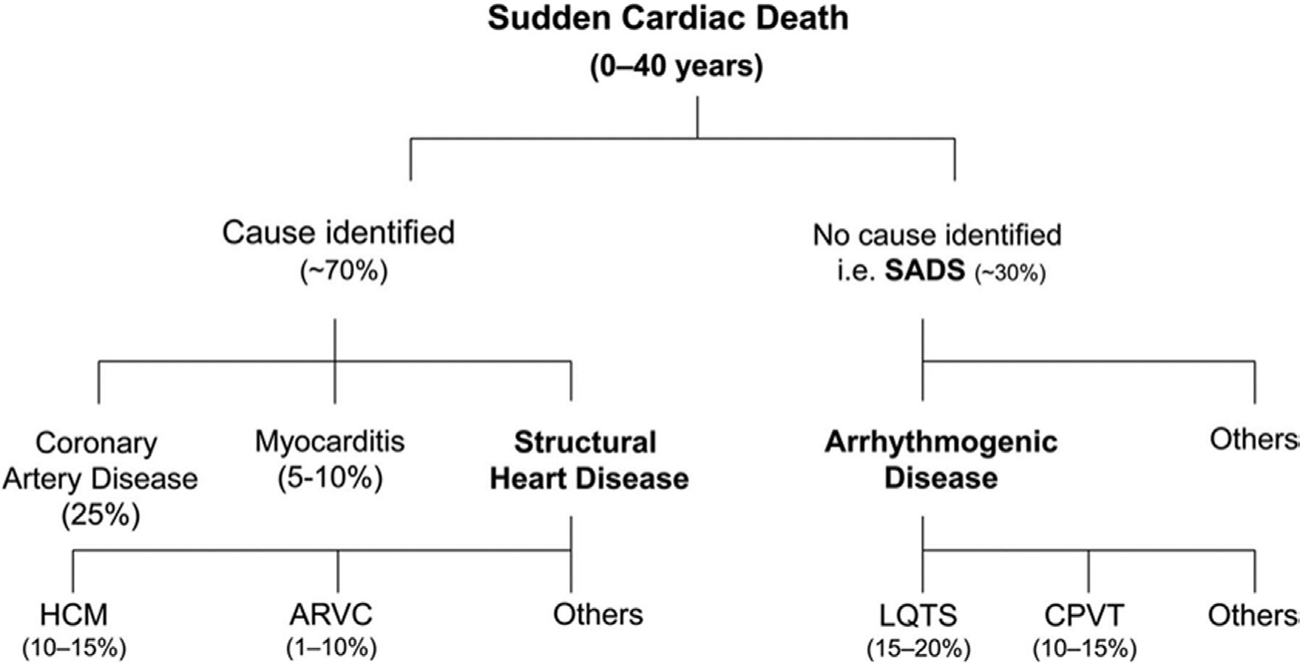
**Overview of causes of sudden cardiac death in the young based on post-mortem studies**. HCM, hypertrophic cardiomyopathy; ARVC, arrhythmogenic right ventricular cardiomyopathy. LQTS, long QT syndrome; CPVT, catecholaminergic polymorphic ventricular tachycardia; SADS, sudden arrhythmic death syndrome. Reprinted from Semsarian et al. (3) with permission of the publisher.

Each year, thousands of individuals die suddenly before the age of 35. Sudden cardiac death (SCD) in this age category has an estimated incidence of 0.005–0.2 per 1000 individuals per year, which is lower than in the general adult population (1). The causes of SCD in the young can be grouped into (1) structural heart disease, where the heart is structurally abnormal and (2) the channelopathies in which the heart is structurally normal (Figure 1) (2). Post-mortem analysis of young SCD cases uncovers a structural cardiac pathology in the majority of cases. However, a subset of around 30% remains unexplained (3). Sudden arrhythmic death syndrome (SADS) is defined as SCD in the setting of a negative autopsy and toxicological analysis (4, 5). In these cases, reaching a diagnosis is challenging and post-mortem genetic testing, the so-called *molecular autopsy*, can crucially contribute to the identification of the underlying (genetic) cause of death (6). This is important for clinical and genetic evaluation of surviving family members that are potentially at risk of SCD (7). The recent advances in sequencing technologies (next-generation sequencing) have made it possible to screen in detail large proportions of the human genome at relatively low cost. However, despite these significant developments, distinguishing true disease-causing genetic variants from the bulk of genetic variation that is not directly associated with the SCD phenotype is of major importance (8). In this review, we will discuss the current achievements of the molecular autopsy in young SADS cases and provide an overview of key challenges in assessing pathogenicity (i.e., causality) of genetic variants identified through next-generation sequencing (NGS).

## The Cardiac Channelopathies

The cardiac channelopathies form a group of inherited disorders associated with the occurrence of arrhythmia and SCD in the presence of a structurally normal heart. These diseases are caused by mutations in genes that encode cardiac ion channel subunits or proteins that regulate and interact with ion channels. The underlying genetic defect leads to cardiac electrical disturbances that have the potential to initiate lethal cardiac arrhythmia (2). The cardiac channelopathies include, among others, the Long QT syndrome (LQTS), the Short QT syndrome (SQTS), Brugada syndrome (BrS), and catecholaminergic polymorphic ventricular tachycardia (CPVT) (5).

### Long QT Syndrome

The LQTS is characterized by prolongation of the QT-interval on the surface electrocardiogram (ECG) associated with syncope and SCD as a result of *torsades des pointes* (TdP) ventricular tachycardia (VT) (9). The disease is genetically heterogeneous and has an estimated prevalence of 1:2000 (10). The inheritance pattern is generally autosomal dominant and mutations in 16 different genes have been associated with the disorder (11). Together, mutations in three major LQTS-causing genes account for ~90% of genotype-positive LQTS patients (7, 12). These genes include *KCNQ1* encoding for the Kv7.1 potassium channel (LQT1, 40–55%), *KCNH2* (LQT2, 30–45%) encoding for the Kv11.1 potassium channel, and *SCN5A* (LQT3, 5–10%) that encodes for the Nav1.5 sodium channel. Genotype–phenotype studies have uncovered genotype-specific clinical presentations that can contribute to the diagnosis of SADS cases based on the circumstances of the SCD (13). In LQT1, cardiac events occur typically during exercise and more specifically during swimming and diving, whereas in LQT2 symptoms are often triggered by sudden auditory stimuli. Patients with LQT3 usually present with symptoms during rest or sleep. The 13 minor LQTS-associated genes have been linked to LQTS in small studies with varying evidence of disease association (2). LQTS can also present with extra-cardiac features. The Jervell and Lange-Nielsen (JLN) syndrome is characterized by significant QTc-interval prolongation accompanied by severe arrhythmias and sensorineural deafness. JLNS is caused by homozygous or compound heterozygous mutations in *KCNQ1* (14) or *KCNE1* (15). The Andersen–Tawil syndrome (LQT7) presents with QTc-interval prolongation, hypokalemic periodic paralysis and facial dysmorphism. The disease is caused by mutations in KCNJ2 (16). Timothy syndrome (LQT8) presents with severe QTc-prolongation, cardiac arrhythmia, syndactyly, autism, and malignant hypoglycemia. The most common associated mutation is the heterozygous G406R mutation in *CACNA1C* (17). The presence of extra-cardiac features has the potential to contribute to the unequivocal identification of the underlying genetic defect and identify an overlooked clinical diagnosis.

### Catecholaminergic Polymorphic Ventricular Tachycardia

Catecholaminergic polymorphic ventricular tachycardia is an inherited arrhythmia syndrome characterized by the onset of life-threatening arrhythmia during exercise or acute emotional stress (18). These patients have a normal resting ECG and the disease can be diagnosed using exercise-stress testing or Holter recording, revealing typical bidirectional or polymorphic VT (5). When left untreated SCD occurs in up to 30% of cases before the age of 40 (19, 20). The autosomal-dominant form of CPVT is caused by mutations in *RYR2* (21) encoding for the ryanodine receptor, whereas an autosomal recessive and more rare form is caused by biallelic mutations in *CASQ2* (22) that encodes for the calsequestrin-2 protein. In addition, mutations in *TRDN*, *CALM1*, *KCNJ2*, and *ANKB* have also been identified in a small set of CPVT patients (2). Mutations in *RYR2* can be identified in ~60% of CPVT cases that have a classical phenotype and these mutations are mainly located in clusters within the gene (21, 23, 24). Genotype-phenotype studies have been conducted and these data suggest a higher arrhythmia risk associated with mutations in the C-terminal portion of the protein (25).

### Brugada Syndrome

Brugada syndrome can present with syncope due to polymorphic VT and SCD as a result of ventricular fibrillation. SCD most commonly occurs during rest or sleep and it typically occurs in males in the fourth decade of life (5, 26). Recent guidelines state that BrS is diagnosed when a coved ST-segment elevation of ≥0.2 mV is present in at least one precordial lead, either occurring spontaneously or after administration of a sodium channel-blocking agent (5). The typical ECG pattern can be concealed and may be intermittently present. In addition to sodium channel blockers, the typical BrS ECG pattern can also be induced by pyrexia (26). Loss-of-function mutations in *SCN5A*, encoding for the Nav1.5 sodium channel, are identified in ~16% of BrS cases (27). In addition to *SCN5A*, multiple other genes have been associated with this disorder (2). Even though the yield of genetic testing is low, genetic testing of *SCN5A* can identify a pathogenic mutation that could contribute to further genetic risk stratification in the family (5, 7). The observation that within some families the *SCN5A* mutation does not segregate with the disease suggests a potential modifying or more complex role for other genetic factors (28). Furthermore, a recent study suggested a more complex form of inheritance for the BrS with an important role for common genetic variation in disease susceptibility (29).

### Short QT Syndrome

The SQTS presents with a short QT-interval on the surface ECG (<350 ms) predisposing to supraventricular and ventricular arrhythmia and is associated with a high risk of SCD (30, 31). The disorder is genetically heterogeneous and inherited in an autosomal-dominant mode. SQTS has been associated with pathogenic variants in genes that encode potassium channels *(KCNQ1, KCNH2*, and *KCNJ2*), which are also implicated in LQTS (32–34). Importantly, SQTS-causing variants in these genes lead to a gain-of-function on the affected channel, whereas the LQTS-causing variants lead to a loss-of-function. In addition, Cav1.2 L-type calcium channel subunits (*CACNA1C*, *CACNB2*) have been associated with SQTS (35). Even though in half of SQTS cases familial disease is present, the yield of genetic testing is around 14% (36).

## The Cardiomyopathies

The inherited cardiomyopathies include hypertrophic cardiomyopathy (HCM), dilated cardiomyopathy (DCM), and arrhythmogenic cardiomyopathy (ACM) (37). The hallmark of HCM is unexplained ventricular hypertrophy, and myocyte disarray and fibrosis during histological analysis (38). The disease has an autosomal-dominant mode of inheritance in the majority of cases, with mutations predominantly located in genes encoding sarcomeric proteins. Most mutations are found in *MYBPC3* and *MYH7* (39, 40). SCD occurs in only a small subset of HCM cases (38). DCM can present with heart failure due to dilatation of the left ventricle and systolic dysfunction (41). In approximately one-third of patients with idiopathic DCM, a positive family history for DCM can be identified (42). The inheritance pattern varies and is most commonly autosomal dominant or autosomal recessive, whereas X-linked inheritance or mitochondrial inheritance is less common (43, 44). The disease is genetically heterogeneous and more than 30 genes have been associated with DCM, although the evidence of disease association is highly variable. The most common genetic causes of DCM are found in *TTN*, *MYH7*, *LMNA*, and *TNNT2* (43). Importantly, mutations in *LMNA* have been associated with a form of DCM with significant cardiac conduction abnormalities and the occurrence of cardiac arrhythmia. Therefore, the identification of a mutation in *LMNA* during molecular autopsy has the potential to offer pre-symptomatic intervention (e.g., implantable defibrillator, pacemaker) to surviving family members carrying the familial *LMNA* mutation (45). ACM is characterized by fibrofatty infiltration of the myocardium and a high susceptibly to ventricular arrhythmia and SCD at young age (46). The disease is most commonly inherited in an autosomal-dominant fashion and gene mutations are mostly found in the following desmosomal genes: *PKP2*, *JUP*, *DSP*, *DSC2*, and *DSG2* (47). ACM has a variable disease expressivity and reduced penetrance among mutation carriers (48). It may affect the right ventricle predominantly (arrhythmogenic right ventricular cardiomyopathy – ARVC), the left ventricle, or both. Genetic testing in ACM can be helpful to identify family members at risk (7).

## The Molecular Autopsy

**Figure 2:**
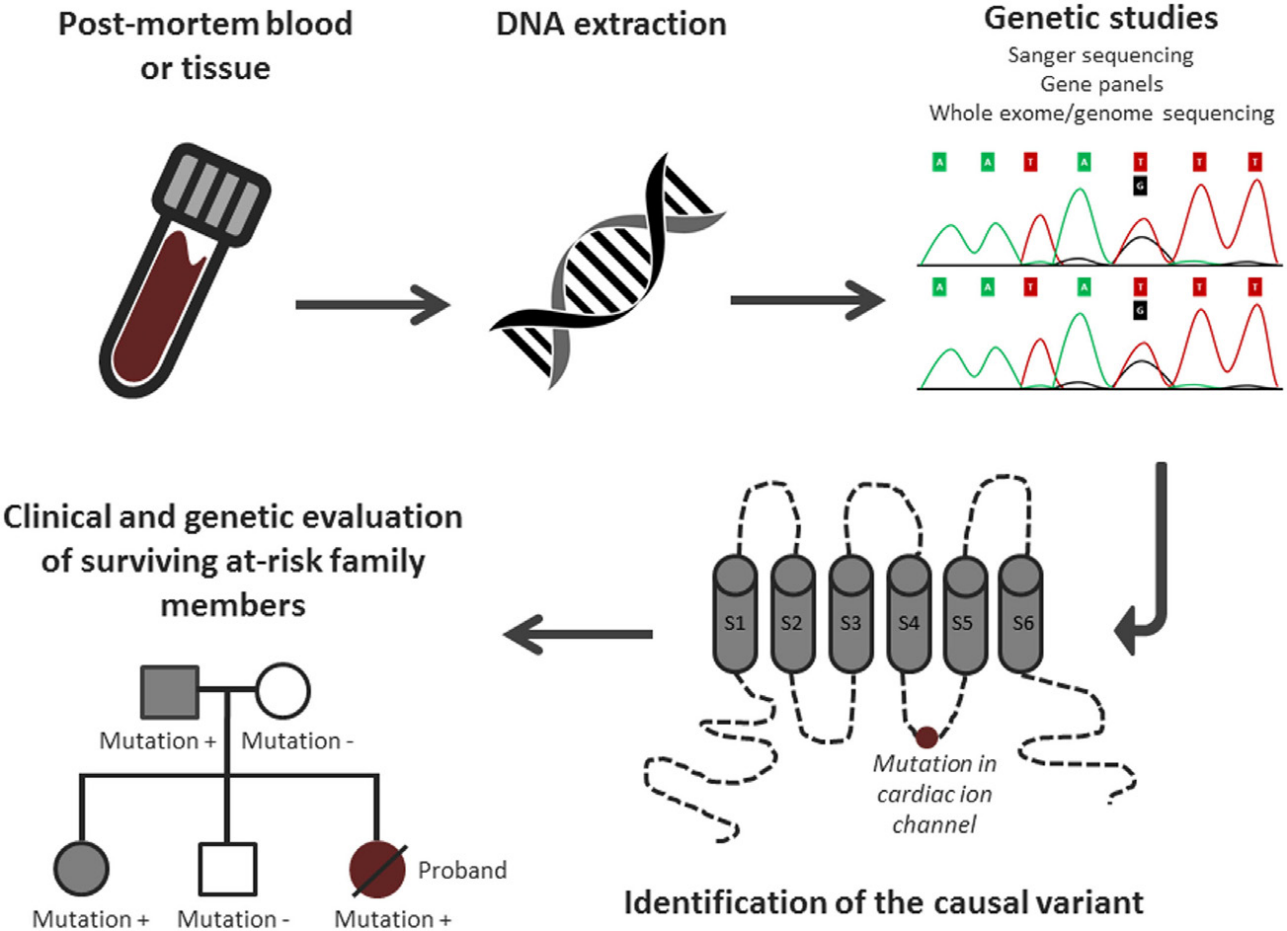
**Overview of the molecular autopsy process**. Adjusted from Semsarian et al. (3) with permission of the publisher.

Post-mortem genetic testing, using DNA extracted from blood or other tissue after death, has an important role in the identification of the underlying genetic cause in SADS cases (i.e., SCD cases with negative toxicology and pathology analysis). This process has been termed the “molecular autopsy” (Figure 2). Recent guidelines recommend the use of post-mortem genetic testing in cases where clinical evidence suggests a diagnosis of the LQTS or CPVT (5, 7).

In 1999, the identification of LQT1 as the underlying cause of death in a 19-year-old female was reported by Ackerman and colleagues (49). Several years after this report, Chugh and colleagues analyzed 5 LQTS-associated genes (*KCNQ1*, *KCNH2*, *SCN5A*, *KCNE1*, and *KCNE2*) in 12 sudden unexplained death cases in whom no diagnosis could be established after thorough post-mortem analysis of 270 adult SCD cases. Through this analysis, the authors identified the same *KCNH2* missense mutation in 2 out of 12 cases (yield of genetic testing: 17%) (50). Shortly afterwards, another study reported the post-mortem genetic analysis in 10 cases of juvenile (13–29 years) sudden unexplained death cases and identified LQTS-associated mutations in two patients (51). Subsequently, multiple similar post-mortem genetic studies have been conducted by several groups (52–58). In one study, 33 young cases were examined for LQT1–6 genes, and a putative pathogenic mutation was identified in 15% of patients (59). Tester and colleagues conducted a post-mortem analysis in 49 cases screening 18 exons of the CPVT-associated gene *RYR2* (60). In a subsequent study in the same cohort, these authors analyzed the three major genes associated with LQTS (*KCNQ1*, *KCNH2*, and *SCN5A*) (61). The genetic yield of CPVT and that of LQTS genetic testing were, respectively, 14 and 20%, with an overall genetic yield reaching 35%. In an extended cohort of 173 autopsy-negative sudden unexplained death cases from the same group, five genes associated with LQTS and *RYR2* were screened (62). In this expanded analysis, 25% out of the 173 cases carried a potentially pathogenic variant in a LQTS-associated gene (14.5%) and *RYR2* (12%). Even though SCD was the presenting event in the majority of these patients, nearly 60% of the mutation positive cases had a family history of cardiac events. These studies showed that a significant proportion of unexplained death in the young is caused by cardiac channelopathies.

## Next-Generation Sequencing Molecular Autopsy Studies

The above-mentioned molecular autopsy studies have investigated a small number of channelopathy-associated genes. Recent advances in sequencing technologies (next-generation sequencing) have now made it possible to screen in detail an increasing number of genes in cardiac gene panels (i.e., >100 genes) at relatively low cost and using a limited amount of DNA. In addition, whole-exome sequencing (WES), where the coding regions of all ~22,000 genes is sequenced, has been introduced in post-mortem genetic testing as well. It is important to note that these NGS-based studies did not only consider more genes, but also extended to the inclusion of genes involved in the inherited cardiomyopathies (in addition to the channelopathy genes). The role of the cardiomyopathy-associated genes in normal-cardiac autopsy SCD cases remains largely unexplored. In evaluating these NGS-based studies, one should keep in mind that they not only screened varying numbers of genes but also employed different methods of variant prioritization (based on minor allele frequency (MAF) in the general population as cut-off, *in silico* prediction tools for variant pathogenicity). Therefore, the genetic yield of these studies should be interpreted in relation to the varying variant curation and categorization. Bagnall and colleagues, conducted a post-mortem WES study in 28 sudden unexplained death cases and identified three rare variants in the major LQTS-associated genes when they focused their analysis on only a small panel of four genes (*KCNQ1*, *KCNH2*, *SCN5A*, and *RYR2*) (63). In subsequent analyses, more than 70 arrhythmia and cardiomyopathy-associated genes were included and this led to the identification of an additional variant in *CACNAC1* that had been previously reported in a LQTS family. Of note, this additional analysis (using a MAF cut-off of <0.1% in 7500 publically available exomes) identified a large number of variants of unknown significance (VUSs), attesting to the complexity of analyzing such data. In a more recent study, WES followed by the analysis of 135 genes associated with cardiac channelopathies and cardiomyopathies was performed in 59 SADS victims (age range: 1–51 years) (64). Of these, 20 cases had subtle post-mortem cardiac structural abnormalities not reaching the diagnostic criteria for one of the cardiomyopathies. A primary analysis using a filtering MAF ≤0.02% based on the NHLBI exome sequencing project identified rare variants in seven probands. Three of these variants were located in ion channel genes of which two were known LQTS-associated *de novo* variants in *SCN5A* and one known CPVT-associated variant in *RYR2*. The other four rare variants were found in cardiomyopathy-associated genes. In a secondary analysis, using a MAF cut-off of 0.02–0.5%, previously reported variants were identified in an additional 10 probands. However, the clinical significance of these variants has yet to be determined.

Recently, Hertz and colleagues screened 52 SCD cases with non-diagnostic structural cardiac abnormalities during autopsy using a gene panel consisting of 100 genes previously associated with cardiac channelopathies and cardiomyopathies (65). Genetic variants were prioritized using MAF in control populations (<1%), measures of evolutionary sequence conservation, prediction of deleteriousness, and prior disease association of the variant in the Human Genome Mutation Database (HGMD). Variants were subsequently classified as (a) likely, (b) unknown, or (c) unlikely to have functional effects by two physicians. Fifteen individuals (29%) were identified as carriers of variants with “likely functional effects” according to their classification system. In another study, Ackerman and colleagues performed WES and gene-specific analysis of 117 sudden death-susceptibility genes in 14 cases of sudden unexplained death in the young (66). In their analyses, eight rare variants in six genes were identified in seven cases. More recently, the same authors performed WES in 21 cases in whom no mutation was found during the screening of *KCNQ1*, *KCNH2*, *SCN5A*, and *RYR2* (67). Interestingly, three variants (*CALM2*-F90L, *CALM2*-N98S and *PKP2*-N634fs) were classified as pathogenic according to the quideline recommendations of the American College of Medical Genetics (ACMG) (68). Of the 18 remaining cases, 7 carried at least 1 VUS in 1 of the 100 genes associated with SCD.

Thus far, several comparable post-mortem genetic studies using NGS have been conducted recently by several groups (69–72). Collectively, from these studies, it is clear that expanding the number of tested genes from small channelopathy panels to large panels containing a broader set of channelopathy genes, and even the cardiomyopathy-associated genes, increases the yield of likely causal variants only slightly as opposed to the large number of VUSs that are uncovered. The interpretation of these variants is challenging and their clinical utility is currently minimal. In addition, the large majority of SADS cases remain unexplained despite NGS screening of large gene panels.

## Implicating Genetic Variants Identified through NGS in the Molecular Autopsy

Post-mortem genetic testing using NGS is plagued by the same issues as genetic testing in patients with aborted SCD (and many other disorders) with the added complication that one cannot undertake further clinical tests in the deceased patient. The incorporation of NGS in post-mortem genetic testing requires the capability of assessing the genetic variants identified. False assignment of causality can have significant consequences for patients and their families (73). Even though assessing pathogenicity (i.e., causality) of genetic variants is complex, there are several steps to aid in this process (74, 68). It is important to note that each of these steps contributes to rather than determines the classification of a given variant.

### Gene-Level Implication

Unlike the major channelopathy or cardiomyopathy-associated genes, some of the minor associated genes have been implicated in disease in small studies and evidence of disease association has not always been robust (absence of linkage data or absence of recurrent implication of the gene in independent families). Including these minor genes in NGS panels often leads to the identification of a plethora of VUSs. Their clinical utility in establishing the diagnosis in a SADS case and for genetic risk stratification of family members is, therefore, likely to be small. Therefore, the evaluation of an identified variant should start with the assessment of the published data linking that gene to a specific form of disease. In addition, these data should also be taken into consideration during the design of clinical channelopathy and cardiomyopathy gene panels.

### Variant-Level Implication

The assessment of a genetic variant has to take into account the large background of genetic variation in the human genome. Healthy individuals carry multiple rare protein-altering variants and this has been described as “genetic background noise.” Consequently, one of the first important steps in variant prioritization is filtering using the variant MAF in the general population using large ancestry-matched publically available reference databases, such as the Exome Aggregation Consortium (WES data from >60,000 individuals) (75). However, rarity of a variant does not, by definition, implicate disease causality.

After the identification of a genetic variant in a SADS case, co-segregation with disease status should whenever possible be performed in surviving family members. *De novo* inheritance of rare genetic variation in an SCD-associated gene in a SADS case, with unaffected parents, provides strong evidence for disease association. Of importance, parental mosaicism, as opposed to *de novo* inheritance should be taken into account during genetic counseling as this could lead to the false assumption that siblings are genetically unaffected. Parental mosaicism has been described previously in Timothy syndrome (LQT8) (76).

The previous identification of the genetic variant in an independent proband displaying the same or similar phenotype is also highly valuable. Such previous associations can be found by scanning the literature and by using in-house or public databases of disease variants. Of importance, these previous published studies should be assessed carefully (i.e., study design, co-segregation in the family, functional data) to assess the strength of disease association. In this regard, some of the previously published “pathogenic” variants in the literature have later been shown to be at such a high MAF in the general population that their role in disease is questioned (77–79). The assessment of a variant’s pathogenicity would benefit from centralized depositories that include curated evidence for previously identified disease-associated variants.

Computational prediction tools, such as sorting intolerant from tolerant (SIFT) and PolyPhen2, can be helpful in the process but should be handled with caution. Measures of evolutionary sequence conservation among species (orthologs) and among proteins derived from same ancestral gene (paralogs) can have value in the assessment of variants. Paralog annotation tools have been applied to the cardiac channelopathies and are freely available online (80). The Grantham score is a measure of the difference in the physicochemical properties of the amino acid substitutions and a higher score indicates larger differences between amino acids (81). Combining these *in silico* prediction tools has been performed for *KCNQ1*, *KCNH2*, and *SCN5A* and has shown a synergistic utility during the assessment of genetic variation within these genes (82, 83). Most of the recently conducted NGS-based port-mortem genetic testing studies have also incorporated *in silico* prediction tools in variant prioritization (65, 69, 70). Despite these developments, prediction algorithms should not be regarded as stand-alone evidence of pathogenicity. Although certain classes of genetic variation, such as splice-site or truncating variants, are much more likely to affect the protein, their role should be assessed in the specific gene context and if loss of function is a known mechanism of disease. Functional studies can contribute to the understanding of a variant’s biological consequences. However, these studies are labor-intensive and require specialized research centers.

## Conclusion and Future Directions

Next-generation sequencing (NGS) has made it possible to screen large gene panels, spanning not only the channelopathy genes but also the cardiomyopathy genes, in search for the cause of SCD. While these panels have made it possible to broadly screen for genetic variation, it comes with the challenge of interpreting any identified VUS. As seen for the cardiac channelopathies and cardiomyopathies, the genetic architecture of SADS is characterized by large genetic and allelic heterogeneity, which adds to the difficulty of genetic screening in these patients. Even though the majority of SADS cases remain elusive after NGS screening, the generated data make it possible to combine similar datasets through future international collaboration. This has the huge potential to demonstrate statistically an excess of rare genetic variation in known SCD genes (or more interestingly in new genes) in comparison to controls through burden testing (74). Even though presumed to be monogenic, the genetic architecture of SADS is largely unknown in the majority of cases and such case-control studies could point toward a genetic model in which an accumulation of rare genetic variation is required to develop symptoms. However, implementation of the oligogenic model in the segregation within families will be challenging and may require different approaches.

## Author Contributions

All authors researched data for the article, discussed its content, and wrote, edited, and reviewed the manuscript.

## Conflict of Interest Statement

The authors declare that the research was conducted in the absence of any commercial or financial relationships that could be construed as a potential conflict of interest.
